# Accessibility and Affordability of Supermarkets: Associations With the DASH Diet

**DOI:** 10.1016/j.amepre.2017.01.044

**Published:** 2017-07

**Authors:** Joreintje D. Mackenbach, Thomas Burgoine, Jeroen Lakerveld, Nita G. Forouhi, Simon J. Griffin, Nicholas J. Wareham, Pablo Monsivais

**Affiliations:** 1 Department of Epidemiology and Biostatistics, Amsterdam Public Health Research Institute, VU Medical Center Amsterdam, Amsterdam, the Netherlands; 2 UKCRC Centre for Diet and Activity Research (CEDAR), MRC Epidemiology Unit, University of Cambridge School of Clinical Medicine, Institute of Metabolic Science, Cambridge, United Kingdom; 3 Primary Care Unit, Department of Health and Primary Care, University of Cambridge, Cambridge, United Kingdom

## Abstract

**Introduction:**

It is unknown whether there is an interplay of affordability (economic accessibility) and proximity (geographic accessibility) of supermarkets in relation to having a Dietary Approaches to Stop Hypertension (DASH)-accordant diet.

**Methods:**

Data (collected: 2005–2015, analyzed: 2016) were from the cross-sectional, population-based Fenland Study cohort: 9,274 adults aged 29–64 years, living in Cambridgeshire, United Kingdom. Dietary quality was evaluated using an index of DASH dietary accordance, based on recorded consumption of foods and beverages in a validated 130-item, semi-quantitative food frequency questionnaire. DASH accordance was defined as a DASH score in the top quintile. Dietary costs (£/day) were estimated by attributing a food price variable to the foods consumed according to the questionnaire. Individuals were classified as having low-, medium-, or high-cost diets. Supermarket affordability was determined based on the cost of a 101-item market basket. Distances between home address to the nearest supermarket (geographic accessibility) and nearest economically-appropriate supermarket (economic accessibility) were divided into tertiles.

**Results:**

Higher-cost diets were more likely to be DASH-accordant. After adjustment for key demographics and exposure to other food outlets, individuals with lowest economic accessibility to supermarkets had lower odds of being DASH-accordant (OR=0.59, 95% CI=0.52, 0.68) than individuals with greatest economic accessibility. This association was stronger than with geographic accessibility alone (OR=0.85, 95% CI=0.74, 0.98).

**Conclusions:**

Results suggest that geographic and economic access to food should be taken into account when considering approaches to promote adherence to healthy diets for the prevention of cardiovascular diseases and other chronic disease.

## Introduction

The consumption of a healthy diet is a priority for reducing obesity and risk of chronic diseases.^1^, ^2^ The Dietary Approaches to Stop Hypertension (DASH) diet emphasizes fruits, vegetables, whole grains, and low-fat dairy products, and is relatively low in red meats and refined carbohydrates.^3^ The DASH diet has shown to lower blood pressure and decrease blood lipids in clinical trials, and is associated with lower risk of cardiovascular disease, including coronary heart disease, stroke, and heart failure.^3^, ^4^, ^5^ Despite recommendations, widespread adoption of, and long-term adherence to, DASH have been limited.

Diet substantially contributes to socioeconomic inequalities in cardiovascular morbidity and mortality.^6^ It is therefore important to get a better understanding of the barriers for adherence to the DASH diet. Reported individual-level barriers include: increased preparation time required to produce healthier meals, low quality of fruits and vegetables available to consumers, perceived lack of availability of (access to) healthy foods, and preferences for foods that are inconsistent with DASH.^7^, ^8^ Healthy, DASH-accordant foods are also perceived to be expensive,^8^ and several studies confirm that adherence to DASH-accordant diets is associated with higher dietary costs.^9^, ^10^

There is also evidence that neighborhood food environments, in particular supermarket access, may be important for diet and health. Cross-sectional observational studies, mostly conducted in the U.S., have found positive associations among the geographic accessibility of supermarkets, diet quality, and weight.^11^, ^12^, ^13^ However, apart from an Irish study showing an association between DASH accordance and supermarket proximity,^14^ studies from outside the U.S. have not found that geographic access plays a significant role in diet and weight.^15^, ^16^

The lack of consistent findings may be due to the importance of economic factors, which are often unaccounted for. Although absence of supermarkets may affect diet and health, where supermarkets are present, economic inaccessibility of healthful foods may still be critical, especially for low socioeconomic groups.^17^, ^18^ Recent studies suggest that taking affordability into account dramatically changes the definition of access to healthy foods.^18^, ^19^ Those with lower SES, or with lower food budgets, often use lower-cost supermarket chains^20^, ^21^ and may have to travel beyond their nearest supermarket to reach an affordable supermarket.^22^ This may partly explain findings from natural experiments that improving geographic access to supermarkets, without addressing economic access, had little impact on dietary behaviors or obesity.^23^, ^24^, ^25^ Yet, empirical evidence on the relative importance of geographic and economic access to supermarkets in relation to individual dietary patterns is scarce.

The aim of this study was to assess the importance of geographic and economic access to supermarkets and their interplay for the DASH diet, hypothesizing that geographic accessibility to supermarkets would be more strongly associated with diet when taking the price of supermarket foods into account.

## Methods

### Study Sample

The Fenland Study is a population-based cohort study of adults born between 1950 and 1975 and registered with general practices in Cambridgeshire, United Kingdom.^26^ The study was conducted by the Medical Research Council Epidemiology Unit. Participants attended one of three testing sites in Cambridgeshire for detailed anthropometric measurements by trained research staff. Participants completed a general lifestyle questionnaire and a food frequency questionnaire (FFQ) for assessment of habitual diet. Exclusion criteria for the Fenland Study included pregnancy, previously diagnosed diabetes, inability to walk unaided, psychosis, or terminal illness. Recruitment for the study started in 2005 and continued until 2015. At the time of this analysis, data on 11,857 participants were available (27% response rate). All study procedures were approved by the Health Research Authority National Research Ethics Service Committee East of England-Cambridge Central, and participants provided written informed consent.

Sex, age, and household size were captured in the Fenland general lifestyle questionnaire. Highest educational attainment was also captured, and stratified into low education (≤11 years of education), medium education (12–13 years of education), and high education (>13 years of education). Household income per year was measured in three groups: <£20,000, £20,000–40,000, and >£40,000. Participants self-reported smoking status as current, former, or never.

### Measures

Participants recorded the frequency and portions of consumption of foods and beverages by completing a validated 130-item, semi-quantitative FFQ.^27^ Overall dietary quality was evaluated using an index of dietary accordance with the DASH diet, adapted from that of Fung et al.^9^, ^28^ The index consists of eight dietary components (grains/grain products, vegetables, fruits, low-fat/fat-free dairy, red and processed meat, nuts/seeds/dry beans, dietary sodium, and foods high in added sugar).^9^ Energy-adjusted residuals^29^ of the eight components were divided into quintiles. By summing individual quintile scores, the overall DASH scores ranged between 8 (least healthy) and 40 (most healthy). DASH accordance was defined as a DASH score in the top quintile (>28 in this cohort).^28^, ^30^

To rank individuals based on their overall dietary costs, individual dietary costs were measured by attributing a food price variable to the foods consumed according to the FFQ. This measure of dietary cost is positively correlated with actual food expenditures, which has been described in detail elsewhere.^31^, ^32^, ^33^ Briefly, retail prices for each of the foods in the FFQ were obtained from five major UK supermarket chains in 2012, which together had a majority market share in that year.^34^ Food prices were combined with the Fenland FFQ food and nutrient database, which allowed for the derivation of dietary costs (£/day, crude diet cost) for each participant. The variable “energy-adjusted dietary costs” was created by energy-adjusting daily (crude) diet costs on the method of residuals,^35^ a standard energy adjustment in epidemiologic studies.^31^ Dietary costs were divided into tertiles, with those in the third tertile having the most expensive diets.

Participants’ home addresses were mapped by postcode using GIS in ArcGIS, version 10. Accurate data on food outlet locations were sourced from ten local councils covering the Cambridgeshire study area in December 2011^36^ and mapped by postcode. The distance between home address and nearest supermarket of any type was calculated along the street network using the Ordnance Survey Integrated Transport Network. This measure of geographic access to supermarkets was divided into tertiles, with those in Tertile 1 having the shortest distance to their nearest supermarket.

Then, supermarkets were classified into one of four economic tiers, based on a 101-item market basket (details described in Appendix Methods and Appendix Table 1, available online). Data on food prices from seven chain supermarkets in Cambridge were collected between December 2013 and January 2014. General Linear Model repeated measures pairwise comparisons identified four supermarket tiers. Given the small number of lowest-cost (*n*=5) and second lowest-cost supermarkets (*n*=6), these categories were combined. Using tertiles of individual dietary costs and the three supermarket cost tiers, individuals with the highest dietary costs were matched to any supermarket, and individuals with the lowest dietary costs were matched with lowest-cost supermarkets. Distance between home address and the nearest economically-matched supermarket was calculated along the street network. This measure of economic access was also divided into tertiles, with those in Tertile 1 having the shortest distance to their nearest economically-matched supermarket. Appendix Figure 2 (available online) illustrates how distance to the nearest supermarket may differ from distance to the nearest economically-matched supermarket.

### Statistical Analysis

Data were analyzed in 2016. Characteristics of individuals in three tertiles of diet cost were assessed using ANOVA and chi-square tests. DASH accordance was compared between men and women, lower and higher education, and lower and higher income.

Associations between tertiles of dietary costs and DASH accordance were examined using multiple logistic regression models. Similarly, associations between tertiles of distance to the nearest and nearest economically-matched supermarket and DASH accordance were examined using multiple logistic regression models. The addition of covariates into the models was theoretically informed a priori and included the following variables: age, sex, highest educational qualification, car ownership, and total energy intake. Associations between tertiles of dietary costs and DASH accordance were additionally adjusted for income and distance to the nearest supermarket. As both Breyer and Voss-Andreae^18^ and Jiao and colleagues^19^ conducted their studies in low-income groups, educational attainment and income were tested for as potential moderators, but evidence for such interaction was not found. In the third model, analyses were additionally adjusted for exposure to other food outlets (restaurants, convenience stores, cafes, entertainment venues selling food, specialist retailers [e.g., butchers, fishmongers], fast food outlets, and other supermarkets) within a 1-mile (1.6-km) radius Euclidean buffer of the nearest supermarket. Adjustment for the availability of other food outlets was to account for food environment “context” as often different types of food outlets (that allow healthy and unhealthy foods to be purchased) are co-located. Sensitivity analyses additionally adjusted for marital status, ethnicity, mode of transport to work, vegetarianism/veganism, alcohol consumption, and year of attendance at the clinical testing site, but this did not affect the results (data not shown).

Given the low prevalence of missing data, complete case analyses were conducted. Those living outside the Cambridgeshire study area (not allowing for supermarket proximity to be calculated, *n*=1,656), participants with extreme values for energy intake based on sex-specific cut offs as suggested by Willett^37^ (*n*=273), and participants with missing data on covariates (*n*=204) were excluded. This resulted in an analytic sample of 9,724 participants (Appendix Figure 3, available online, provides a participant flow chart). The analytic sample was slightly younger (aged 48.2 years vs 49.5 years, *p*<0.001) and less likely to be male (48.1% vs 51.9%, *p*=0.001), but did not differ in terms of DASH scores (24.0 vs 24.0, *p*=0.97) compared to the full sample. A two-sided α level of 0.05 was used to test for statistical significance. All analyses were conducted using SPSS, version 22.

## Results

Mean (SD) age of the participants was 48.2 (7.3) years and 48.1% were men (Table 1). The mean (SD) estimated dietary costs were £4.21 (£1.25)/day. Participants with lower dietary costs had lower incomes and lower educational attainment and were less likely to have DASH-accordant diets, relative to participants with higher dietary costs. Mean distance to any supermarket was similar for those with low and high dietary costs (3.8 km), but mean distance to the nearest economically-matched supermarket was substantially larger for those with low dietary costs (10.8 km). Overall, 17.3% of the sample consumed diets that were DASH-accordant (DASH score >28), with the percentage higher for women than men (22.1% vs 12.1%, respectively). Individuals with lower income and lower educational attainment were less likely to have DASH-accordant diets (Table 2). Individuals with DASH-accordant diets had lower prevalence of hypertension and lower mean BMI compared with those with less DASH-accordant diets (Appendix Table 2, available online).

**Table 1:** Characteristics of Individuals Across Tertiles of Energy-Adjusted Dietary Costs in the Fenland Study (*n*=9,724)

**Variable**	**Dietary costs**			
	**T1 (*****n*****=3,241)**	**T2 (*****n*****=3,242)**	**T3 (*****n*****=3,241)**	**Overall (*****n*****=9,724)**
Age, years, M (SD)	47.3 (7.3)	48.0 (7.2)	49.2 (7.2)	48.2 (7.3)
Sex (% male)	58.1	46.6	39.5	48.1
Dietary costs, £^a^/day, M (SD)	3.40 (0.91)	4.02 (0.85)	5.22 (1.19)	4.21 (1.25)
Household income, <£20,000^a^/year, %	14.1	10.9	10.3	11.7
Educational attainment, ≤11 years of education, %	23.0	19.1	19.7	20.6
Smoking, current smoker, %	13.2	10.4	11.6	11.7
Car ownership, %	92.0	94.6	95.3	94.0
Fruit intake, g/day, M (SD)	175.3 (135.2)	233.3 (156.0)	303.6 (259.7)	246.4 (202.0)
Vegetable intake, g/day, M (SD)	206.3 (93.3)	273.0 (107.2)	365.3 (177.3)	280.0 (145.1)
Energy intake, kcal/day, M (SD)	1,971 (597)	1,859 (529)	1,952 (537)	1,927 (557)
DASH score,^b^ M (SD)	22.7 (4.5)	24.0 (4.5)	25.3 (4.5)	24.0 (4.7)
Achieving DASH accordance,^c^ %	10.0	17.4	24.5	17.3
Geographic access supermarket,^d^ km, M (SD)	3.8 (3.6)	3.8 (3.7)	3.8 (3.6)	3.8 (3.6)
Economic access supermarket,^e^ km, M (SD)	10.8 (8.9)	5.1 (3.9)	3.8 (3.6)	6.6 (6.7)

a In 2012, GBP£1=USD$1.61.

b DASH scores range from 8 to 40.

c DASH score of >28 (fifth quintile); T1=tertile 1 (lowest dietary costs) and T3=tertile 3 (highest dietary costs).

d Defined as street network distance to the nearest supermarket of any type.

e Defined as street network distance to the nearest economically-matched supermarket, based on supermarket price and dietary costs. DASH, Dietary Approaches to Stop Hypertension; T, tertile.

**Table 2:** DASH Accordance by Sex, Educational Attainment, and Household Income in the Fenland Study (*n*=9,724)

**Variable**	***n*****(%)**	**DASH score, M (SD)**	**% DASH-accordant**^a^	**Achieving DASH accordance**	
				**OR (95% CI)**	***p*****-value**
Sex					
Male	4,673 (48.1)	23.0 (4.6)	12.1	**0.47** (0.42, 0.52)	<0.001
Female	5,051 (51.9)	25.0 (4.5)	22.1	1	
Household income^b^					
<£20,000 per year	1,119 (11.7)	23.5 (4.9)	17.7	**0.83** (0.70, 0.98)	0.030
£20,000–40,000 per year	3,409 (35.8)	23.7 (4.7)	16.4	**0.83** (0.74, 0.94)	0.002
>£40,000 per year	5,002 (52.5)	24.3 (4.5)	17.9	1	
Educational attainment					
≤11 years of education	2,004 (20.6)	23.1 (4.6)	12.8	**0.43** (0.37, 0.51)	<0.001
12–13 years of education	4,571 (47.0)	23.6 (4.7)	15.5	**0.59** (0.53, 0.66)	<0.001
>13 years of education	3,149 (32.4)	25.2 (4.4)	22.8	1	

*Note:* Boldface indicates statistical significance (*p*<0.05). ORs for sex are adjusted for age and educational attainment; ORs for income and educational attainment are adjusted for age and sex.

a DASH score >28 (fifth quintile).

b In 2012, GBP£1=USD$1.61. DASH, Dietary Approaches to Stop Hypertension.

Table 3 shows that, after maximum covariate adjustment (Model 4), individuals in the middle and lowest tertile of dietary costs had 29% (95% CI=0.62, 0.81) and 58% (95% CI=0.36, 0.49) lower odds of having a DASH-accordant diet.

**Table 3:** Energy-Adjusted Dietary Costs Associated With Likelihood of Having a DASH-Accordant Diet (*n*=9,724)

**Exposure measure**	**Model 1**	**Model 2**	**Model 3**	**Model 4**
Dietary costs				
T1	**0.34** (0.30, 0.40)	**0.40** (0.35, 0.46)	**0.40** (0.35, 0.46)	**0.40** (0.35, 0.46)
T2	**0.65** (0.58, 0.73)	**0.70** (0.62, 0.79)	**0.71** (0.63, 0.80)	**0.71** (0.63, 0.81)
T3	1	1	1	1

*Note:* Values are OR (95% CI). Achieving a DASH-accordant diet was defined as a DASH score >28. Coefficients were derived from logistic regression analyses. Boldface indicates statistical significance (*p*<0.05). T1 is the tertile with lowest dietary costs, while T3 is the tertile with highest dietary costs (reference group). Model 1 is an unadjusted model. In Model 2 associations are adjusted for individual-level covariates (age, sex, educational level, and energy intake). In Model 3, associations are additionally adjusted for income. In Model 4, associations are additionally adjusted for distance to the nearest supermarket.

DASH, Dietary Approaches to Stop Hypertension; T, tertile.

Table 4 presents the odds of having a DASH-accordant diet according to distance to the nearest and nearest economically-matched supermarket. In the unadjusted model (Model 1), individuals living farthest away from their nearest supermarket had 24% lower odds of having a DASH-accordant diet (OR=0.76, 95% CI=0.66, 0.86), relative to those living closest, with evidence of a dose−response association. Adjustment for sociodemographic factors (Model 2) attenuated the association to non-significance for individuals in Tertile 2. Additional adjustment for exposure to other food outlets attenuated the association further (Model 3), but those living farthest away from their nearest supermarket still had 15% lower odds of having a DASH-accordant diet (OR=0.85, 95% CI=0.74, 0.98), relative to those living closest.

**Table 4:** Geographic and Economic Supermarket Access in Relation to Likelihood of Having a DASH-Accordant Diet (*n*=9,724)

**Exposure measure**	**Model 1**	**Model 2**	**Model 3**
Geographic access to supermarkets			
T1 (ref; 0–1.12 km)	1	1	1
T2 (1.13–5.00 km)	**0.83** (0.73, 0.94)	0.93 (0.82, 1.07)	0.96 (0.84, 1.10)
T3 (5.01–15.08 km)	**0.76** (0.66, 0.86)	**0.81** (0.71, 0.93)	**0.85** (0.74, 0.98)
Economic access to supermarkets			
T1 (ref; 0–2.03 km)	1	1	1
T2 (2.04–7.35 km)	**0.67** (0.59, 0.76)	**0.71** (0.63, 0.81)	**0.73** (0.64, 0.83)
T3 (7.36–32.16 km)	**0.53** (0.46, 0.60)	**0.57** (0.50, 0.66)	**0.59** (0.52, 0.68)

*Note:* Values are OR (95% CI). Accordance to DASH was defined as a DASH score >28. Coefficients were derived from logistic regression analyses. Boldface indicates statistical significance (*p*<0.05). T1 is the tertile with shortest distance to the nearest supermarket (reference group), while T3 is the tertile with the longest distance to the nearest supermarket. Model 1 is an unadjusted model; in Model 2 associations are adjusted for individual-level covariates (age, sex, car ownership, educational level, and energy intake); in Model 3 associations are additionally adjusted for exposure to other food outlets within a 1-mile Euclidean buffer of the nearest supermarket.

DASH, Dietary Approaches to Stop Hypertension; T, tertile.

Similar associations, but with stronger effect sizes, were observed for distance to the nearest economically-matched supermarket. In the unadjusted model (Model 1), individuals living farthest away from their nearest economically-matched supermarket had 47% lower odds of having a DASH-accordant diet (OR=0.53, 95% CI=0.46, 0.60), relative to those living closest. Additional adjustment for sociodemographic factors and exposure to other food outlets (Model 3) showed that individuals living farthest away from their nearest economically-matched supermarket still had 41% lower odds of having a DASH-accordant diet (OR=0.59, 95% CI=0.52, 0.68), compared with individuals living closest, again with evidence of a dose−response association. Coefficients for other covariates included in these models are presented in Appendix Tables 3 and 4 (available online).

Sensitivity analyses, adjusting for a variety of other factors (Methods), did not materially affect the results (data not shown).

## Discussion

Given the beneficial effects of the DASH diet on risk of hypertension, heart failure, and coronary heart disease,^3^, ^4^, ^5^ understanding the barriers in adherence to this diet is of public health importance. This population-based UK study demonstrated that the likelihood of consuming a DASH-accordant diet was dependent on both economic and geographic factors. The finding that both the local food environment and individual dietary budgets contribute to the accessibility of healthy food options suggests that improving adherence to a DASH diet may require structural approaches that take into account both affordability and proximity.

As suggested previously, economic factors such as dietary costs were found to be strongly associated with diet quality.^9^ Having low dietary costs was associated with a 58% lower likelihood of consuming a DASH-accordant diet, comparable to the odds in participants with lowest education. Taking into account distance to the nearest supermarket did not influence these estimates, concordant with research that showed when modeled jointly, only price and not distance to stores was important for diet.^22^

Studying the importance of geographic accessibility of supermarkets revealed that individuals living farthest away from any supermarket were 15% less likely to consume DASH-accordant diets. This finding is in agreement with previous studies showing associations of supermarket proximity with dietary quality generally,^11^, ^38^ and accordance to DASH in particular^14^ but for the first time shown in a UK context.

Finally, supermarket access was defined according to both economic and geographic considerations, using a more nuanced metric consistent with theory described in two U.S. studies,^18^, ^19^ and for the first time in relation to dietary quality in the United Kingdom. As hypothesized, economic accessibility of supermarkets was more strongly related to dietary quality than geographic accessibility alone, with those living farthest away from an economically-matched supermarket having 41% lower likelihood of having a DASH-accordant diet. As such, price differences may be making some supermarkets more or less accessible for some than others, as a function of individual food budgets.^19^ By taking into account economics, latent (otherwise invisible) barriers to the uptake and maintenance of a DASH-accordant diet may have been captured that have not been captured in previous geographic studies.

### Implications

Enhancing geographic access to supermarkets may constitute an effective supply-side solution to improving poor diets and health,^39^, ^40^ as international research indicates that proximity to supermarkets remains highly variable.^41^ However, as shown in a number of natural experiments, improving geographic access to supermarkets alone may be insufficient to promote behavior change.^23^, ^24^, ^25^ Public health gains could be maximized through neighborhood-level interventions focused on healthy and affordable food retail access, which is likely to be especially important for price-sensitive low-income consumers.

As affordability depends on both food price and food budgets, accessibility of healthy foods should take into account the purchasing power of individuals. Demand-side solutions could be in the form of financial incentives for the purchase of healthy foods.^42^ As an example of supply- and demand-side interventions in parallel, U.S. farmers markets established to provide healthy food retail in the absence of supermarkets are also increasingly accepting Supplemental Nutrition Assistance Program coupons from low-income residents.^43^ To prevent widening of socioeconomic inequalities in diet, it may be important to target such food price policies specifically at low socioeconomic groups.^44^

### Limitations

A number of factors may limit this study. Dietary intakes and dietary cost estimates were derived from an FFQ, an instrument subject to error and known biases.^37^ This study lacked information on participants’ actual food spending and the origin of foods consumed, which could have resulted in misclassification of dietary cost. However, dietary costs (as derived with reported dietary intakes and a fixed database of food prices) are modestly but positively correlated with actual food spending,^31^, ^32^, ^33^ therefore, being suitable for our purpose of ranking individuals into tertiles of dietary cost. There was no information on the actual shopping location of the participants. Instead, access to supermarkets was defined as proximity, based on the decreased likelihood for environments to influence individuals as a function of distance.^45^ As the identification of supermarket tiers was based on full retail product prices, and collected over 2 consecutive months, supermarkets with more discounts on average, and at this time of year, may have been misclassified. Combining this with a lack of information on actual spending, participants may have been mismatched to supermarket tiers. Lastly, further exposure misclassification may have resulted from: calculating supermarket proximity only from the home address; neglecting other environmental settings; and temporal mismatch, as exposures (supermarket locations, 2011) were only measured at one time point, whereas outcomes (diet, 2005–2014) were measured over 9 years.

This study did, however, benefit from the combination of objective information on the geographic location of supermarkets, the costs of food in these supermarkets on the basis of a 101-item market basket, and detailed information on the diet quality of more than 9,000 UK adults. Although originating from the U.S., the DASH diet has shown to be congruent with UK food preferences.^46^ A further strength is the derivation of individual-level dietary costs as a measure of economic access, providing an insight into the monetary value of individuals’ diets. Incorporating economic accessibility into our measure of physical supermarket access allowed for a more comprehensive definition of access. Finally, adjusting the analyses for exposure to other food outlets controlled for food environment “context,” specifically allowing for the fact that many healthy and unhealthy foods can be purchased at other food outlets.

## Conclusions

This study provides novel evidence that geographic access to supermarkets is particularly important for accordance to the DASH diet once economic access to food is taken into account. The fact that higher dietary costs and supermarket proximity were associated with DASH accordance suggests that interventions to improve healthy eating should include structural changes involving both affordability and proximity of healthy food options.
