# Supplementary material for: Accessibility and Affordability of Supermarkets: Associations With the DASH Diet
Source: Am J Prev Med. 2017 Jul;53(1):55–62. doi: 10.1016/j.amepre.2017.01.044 (PMC5478361; doi:10.1016/j.amepre.2017.01.044)
Supplement: Supplementary file 1 — Supplementary material [file mmc1.pdf]

Appendix

Appendix Methods - Characterization of supermarkets according to cost

All supermarkets were classified into one of four economic tiers, based on a 101-item market basket. The market basket should reflect both health and unhealthy items and contain foods as used in the Fenland food frequency questionnaire. The purpose was for the market basket to provide between-supermarket heterogeneity in food prices.

A number of food lists were used as input for the construction of the market basket: the EPIC FFQ food groups (on which the Fenland FFQ was based); the food groups from the 'eat well' plate[1]; the 10 major food groups according to the European Food Safety Authority (EFSA)[2]; nutrients and foods with established relationship to health (EFSA); seven foods common in the European food-based-dietary guidelines[3]; food groups in the Centre for Public Health Nutrition (CPHN) market basket[45]; food items in the UK CPI market basket[6]. On the basis of these lists we chose 101 items, which were categorized into the following 11 food groups: fruits, vegetables, fat, dairy, breads/cereals, rice/pasta/potatoes, fish, meat, sugars and sweets, beverages and 'other'. Amounts/units per item were chosen based on the UK CPI market basket.

Data were collected on each item in the market basket in each of the seven supermarket chains in the area between December 2013 and January 2014 via www.mysupermarket.co.uk (an online price comparison website for UK supermarkets). The locations of all geocoded supermarkets in the study area are shown in **Appendix Figure 1**. Prices for Morrison's were collected in-store. If not all supermarkets sold all products, we imputed missing values using mean imputation. We then compared the total price of the market basket as well as the price of items in the 12 food groups were compared between supermarket chains. We performed General Linear Model (GLM) repeated measures analysis to examine whether the price of the market basket was different between the seven supermarket chains. We used pairwise comparisons to examine which supermarket chains significantly differed in average market basket price. We additionally tested whether the prices of food items within food groups differed by supermarket brand using interaction terms between supermarket brand and food group. Analyses were conducted using SPSS version 22.

In **Appendix Table 1** we present the description of market basket food items (with their units) by food category, and their prices for each of the seven supermarket chains. Not all products were available in all supermarkets, such that only 73 items were available in all seven supermarket chains. Missing values were therefore imputed. The cost of the 101-item market basket of the most expensive supermarket (Co-Op; £355) was approximately £132

---

[1] http://www.nhs.uk/Livewell/Goodfood/Pages/eatwell-plate.aspx

[2] http://www.efsa.europa.eu/en/datexfoodcdb/datexfooddb.htm

[3] http://www.eufic.org/article/en/expid/food-based-dietary-guidelines-in-europe/

[4] http://depts.washington.edu/uwcphn/reports/brf2_prices.pdf

[5] http://depts.washington.edu/uwcphn/reports/cphnbrf2.pdf

[6] http://data.gov.uk/dataset/cpi_and_rpi_basket_of_goods_and_services

more expensive than of the cheapest supermarket (Aldi; £223). General Linear Model (GLM) repeated measures analysis confirmed that the prices of 101 food items were different between the seven supermarket chains (Lower-bound F=15.03, p<0,001). We additionally tested whether prices of food items within food groups differed per supermarket brand, but the interaction between outlet and food group showed that this was not the case (Lower-bound F=1.375, p=0.199). (The results were similar when using only prices of complete cases, i.e., comparing prices of the 73 products that were available in all supermarkets.)

Using Pairwise comparisons we were able to discriminate between supermarkets that were significantly indistinguishable in terms of price and supermarkets that significantly differed from each other. Aldi and Asda each significantly differed from all other supermarkets, while Sainsbury’s, Tesco and Morrison’s had similar price levels, just like Waitrose and Co-Op. This suggests that there were four tiers of supermarkets, of which Aldi comprised the lowest tier, Asda comprised the second lowest tier, Sainsbury’s, Tesco and Morrison’s comprised the third tier, and Waitrose and Co-Op comprised the most expensive tier. In the Cambridgeshire area, 31 medium priced supermarkets were present, and 41 supermarkets in the most expensive tier. Given the small number of lowest cost (n=5) and second lowest cost supermarkets (n=6) in the area we combined the two lowest categories into one.

Appendix Table 1. Description of food items (unit) by food category and their prices in each of the seven supermarket chains.

Food categories and food items	Sainsbury's	Tesco	Waitrose	ALDI	ASDA	Morrison's	Co-Op
**Fruit:** Apples – Granny Smith (kg), pears - Conference (kg), bananas (kg), grapes (kg), oranges (1p), grapefruits (1p), kiwi (1p), sultanas – dried (0.5 kg), prunes – dried (0.5 kg), strawberries – fresh (kg), apricots - dried (0.5 kg)	£23.97	£27.55	£28.48	£19.19	£23.15	£25.24	£34.89
**Vegetables:** cucumber (each), tomatoes (kg), carrots (kg), cabbage – white (kg), onions - red (kg), mushrooms – cup (kg), pre-packed salad (0.1-0.25 kg), broccoli (kg), courgette (kg), spinach (kg, fresh), beansprouts - fresh (0.2 kg), red pepper (each)	£22.57	£20.43	£21.41	£19.42	£17.39	£21.22	£32.41
**Fat:** salted butter (0.25 kg), margarine (0.5 kg), olive oil (l), sunflower oil (l)	£9.29	£9.43	£8.14	£5.40	£5.71	£5.60	£3.50
**Dairy products:** cheddar cheese (0.35 kg), cheese spread (0.2 kg), parmesan cheese (kg), milk - semi-skimmed (4p), milk – whole (4p), low fat natural yoghurt (0.5 kg), powdered baby formula (0.9 kg), double cream – fresh (0.1 kg), ice cream – vanilla (l)	£46.80	£43.19	£54.07	£31.24	£36.60	£40.61	£45.99
**Bread/cereals:** loaf white bread – large (1p), loaf wholemeal bread – large (1p), bread rolls – brown/white (6p), cereal bar (6-10p), muesli (kg), garlic bread (1p), breakfast cereal – cornflakes (kg)	£9.27	£9.22	£12.56	£8.10	£9.87	£8.70	£13.07
**Rice/potatoes/pasta:** dry spaghetti (0.5 kg), basmati rice (kg), potatoes (kg)	£3.23	£3.06	£3.55	£2.03	£2.67	£3.74	£3.61
**Fish:** white fish fillets (kg), salmon fillets (kg), canned tuna (0.2 kg), frozen fish fingers (8-12p), frozen prawns (kg)	£41.89	£36.16	£55.38	£30.76	£32.93	£42.74	£48.58

Appendix Table 1 (Continued).

Food categories and food items (continued)	**Sainsbury's**	**Tesco**	**Waitrose**	**ALDI**	**ASDA**	**Morrison's**	**Co-Op**
**Meat:** frozen beefburgers (4p), cooked ham (0.1 kg), cooked turkey (0.1 kg), bacon (kg), lean beef mince (kg), lamb leg (kg), chicken – whole (kg), chicken breast (kg), beef steak (kg), lamb chops (kg), eggs – medium (dozen)	£63.62	£71.89	£77.93	£62.70	£70.97	£71.61	£90.70
**Sugars and sweets:** milk chocolate (0.1 kg), mars bar (4p), twix bar (4p), dark chocolate (0.1 kg), chewing/bubble gum (5x 10p), mints (0.1 kg), bag of sweets (0.15-0.25 kg), digestive biscuit (0.5 kg), lemon sponge (1p), doughnut (1p), cakes – chocolate (5-6p)	£11.30	£10.71	£12.22	£8.02	£9.55	£10.55	£17.35
**Beverages:** cola (2 l), beer – Becks (0.6 l), tea bags (80p), coffee – filter (0.1 kg), hot chocolate (0.4 kg), pure orange juice (1 l), pure apple juice (1 l), apple cider (0.5 l), still water (2 l), rosé wine (0.75 l), white wine (0.75 l), red wine (0.75 l)	£23.44	£22.76	£25.96	£20.14	£21.03	£25.06	£34.85
**Ready meals:** tomato soup (0.4 l), beef lasagne - fresh (0.4 kg), pizza – frozen (0.3-0.45 kg), mash and sausage – fresh (1p), pasta chicken - fresh (1p), beef ravioli – fresh (1p), Indian butter chicken – fresh (1p)	£12.15	£12.94	£16.24	£10.91	£11.09	£12.22	£18.69
**Other:** light mayonnaise (0.5 kg), tomato ketchup (0.1 l), tofu (0.1 kg), frying chips – frozen (kg), oven chips – frozen (kg), raspberry jam (0.35-0.45 kg), cashew nuts – salted (0.2 kg), peanuts – salted (0.2 kg), flour (1.5 kg)	£7.63	£7.65	£9.99	£5.60	£6.33	£11.85	£11.19
**Total market basket price:**	**£275.16**	**£274.99**	**£325.93**	**£223.51**	**£247.29**	**£279.14**	**£354.83**

In 2012, GBP £1 = USD $1.61. kg, kilogram. l, liter. p, piece.

Appendix Table 2. Characteristics of individuals across quintiles (range) of Dietary Approaches to Stop Hypertension (DASH) accordance in the Fenland Study (N=9724).

Variable of interest	Q1 (9-19) n=1696	Q2 (20-22) n=1971	Q3 (23-24) n=2366	Q4 (25-27) n=2010	Q5 (28-39) n=1681	Total sample n=9724
Age - years	47.0 (7.4)	47.9 (7.2)	48.1 (7.2)	48.6 (7.3)	49.1 (7.3)	48.2 (7.3)
Sex (% men)	63.7%	56.3%	47.8%	39.1%	33.6%	48.1%
Household income (% <£20,000 per year)*	14.5%	13.3%	10.5%	9.1%	12.0%	11.5%
Educational attainment (% ≤11 years of education)	27.4%	21.9%	20.3%	18.4%	15.3%	20.6%
Employment status (% currently employed)	90.7%	90.6%	91.2%	88.8%	88.7%	90.0%
Marital status (% married or cohabiting)	81%	82.7%	81.6%	83.1%	81.0%	81.9%
Ethnicity (% White)	97.5%	97.6%	96.6%	96.0%	95.3%	96.6%
Smoking (% current smoker)	20.5%	14.6%	11.2%	6.9%	6.2%	11.7%
Alcohol consumption (% never or less than once a month)	15.9%	13.7%	14.6%	13.9%	17.3%	15.0%
Car ownership (%)	93.5%	94.9%	93.9%	94.7%	94.0%	94.0%
Main mode of transport to work (% car)†	81.0%	77.5%	74.5%	74.1%	75.1%	64.7%
Energy intake - kcal/day	2027 (606)	1930 (571)	1871 (551)	1881 (523)	1958 (557)	1927 (557)
Vegan or vegetarian (% current)	0.4%	1.1%	2.8%	6.8%	15.2%	5.0%
Dietary costs - £/2000 kcal	4.11 (0.93)	4.31 (0.95)	4.51 (1.02)	4.63 (1.06)	4.77 (1.01)	4.47 (1.02)
Body mass index - kg/m^2‡	27.5 (4.9)	27.2 (4.6)	27.0 (4.8)	26.5 (4.6)	25.9 (4.5)	26.9 (4.2)
Hypertension (%)¥	7.0%	6.4%	5.1%	4.8%	4.0%	5.4%
OR (95%CI) for being hypertensive§	reference	0.91 (0.69; 1.20)	0.78 (0.58; 1.03)	0.78 (0.57; 1.05)	0.73 (0.52; 1.02)	-

Numbers are means (SD), percentages or Odds Ratios (95%CI). Q1 = quintile 1 (lowest DASH score). Q5 = quintile 5 (highest DASH score).
* In 2012, GBP £1 = USD $1.61.
† Percentage of participants indicating that they commuted by car always or usually.
‡ Participants were measured barefoot and wore light clothing. Weight was measured to the nearest 200 g with a calibrated electronic scale (TANITA model BC-418 MA; Tanita, Tokyo, Japan). Height was assessed to the nearest 0.1 cm with a wall- mounted stadiometer (SECA 240; Seca, Birmingham, United Kingdom). Body mass index (BMI; in kg/m^2) was calculated as weight divided by square height.
¥ Defined as having a systolic blood pressure ≥ 140 and a diastolic blood pressure ≥ 90, or currently receiving pharmaceutical treatment for hypertension.
§Odds Ratio for being hypertensive compared to individuals in the first DASH quintile. Adjusted for age, sex, educational attainment, dietary energy intake, alcohol consumption, body mass index, physical activity energy expenditure and smoking status.

Appendix Table 3. Associations of tertiles of energy-adjusted dietary costs with likelihood of having a Dietary Approaches to Stop Hypertension (DASH)-accordant diet (N=9724) – all covariates presented.

	Model 1		Model 2		Model 3		Model 4	
Exposure measure	OR	95%CI	OR	95%CI	OR	95%CI	OR	95%CI
Daily dietary costs								
T1	**0.34**	0.30; 0.40	**0.40**	0.35; 0.46	**0.40**	0.35; 0.46	**0.40**	0.35; 0.46
T2	**0.65**	0.58; 0.73	**0.70**	0.62; 0.79	**0.71**	0.63; 0.80	**0.71**	0.63; 0.81
T3	1		1		1		1	
Age			**1.03**	1.02; 1.03	**1.02**	1.01; 1.03	**1.02**	1.01; 1.03
Sex			**2.08**	1.85; 2.34	**2.07**	1.83; 2.36	**2.09**	1.84; 2.37
Education								
≤11 years of education			**0.44**	0.38; 0.52	**0.40**	0.33; 0.48	**0.41**	0.52; 0.68
12-13 years of education			**0.58**	0.52; 0.65	**0.57**	0.50; 0.66	**0.59**	0.34; 0.49
>13 years of education			-		-		-	
Energy intake (kcal/day)			**1.00**	1.00; 1.00	**1.00**	1.00; 1.00	**1.00**	1.00; 1.00
Household composition					0.97	0.91; 1.02	0.97	0.92; 1.03
Household income								
<£20,000 per year					-		-	
£20,000 – 40,000 per year					1.11	0.92; 1.42	1.10	0.96; 1.26
>£40,000 per year					1.14	0.96; 1.27	1.14	0.92; 1.41
Geographic access to supermarkets (km)							**0.97**	0.96; 0.99

DASH, Dietary Approaches to Stop Hypertension. Accordance to DASH was defined as a DASH score >28. Coefficients were derived from logistic regression analyses. **Boldface** indicates statistical significance (p<0.05). T1 is the tertile with lowest dietary costs, while T3 is the tertile with highest dietary costs (reference group). Model 1 is an unadjusted model. In model 2 associations are adjusted for individual level covariates (age, sex, educational level and energy intake). In model 3 associations are additionally adjusted for income. In model 4, associations are additionally adjusted for distance to the nearest supermarket.

Appendix Table 4. Associations of geographic and economic access to supermarkets with likelihood of having a Dietary Approaches to Stop Hypertension (DASH)-accordant diet (N=9724) – all covariates presented.

	Model 1		Model 2		Model 3	
Exposure measure	OR	95%CI	OR	95%CI	OR	95%CI
**Geographic access to supermarkets**						
T1 (ref; 0-1.12 km)	1		1		1	
T2 (1.13-5.00 km)	**0.83**	0.73; 0.94	0.93	0.82; 1.07	0.96	0.84; 1.10
T3 (5.01-15.08 km)	**0.76**	0.66; 0.86	**0.81**	0.71; 0.93	**0.85**	0.74; 0.98
Age			**1.03**	1.02; 1.04	**1.03**	1.02; 1.04
Sex			**2.30**	2.05; 2.57	**2.30**	2.05; 2.58
Education						
≤11 years of education			**0.44**	0.37; 0.51	**0.45**	0.38; 0.53
12-13 years of education			**0.60**	0.54; 0.68	**0.62**	0.55; 0.70
>13 years of education			**-**			
Car ownership			0.84	0.68; 1.04	0.87	0.70; 1.08
Energy intake (kcal)			**1.00**	1.00; 1.00	**1.00**	1.00; 1.00
Presence of other food outlets					**1.00**	1.00; 1.00
**Economic access to supermarkets**						
T1 (ref; 0-2.03 km)	1		1		1	
T2 (2.04-7.35 km)	**0.67**	0.59; 0.76	**0.71**	0.63; 0.81	**0.73**	0.64; 0.83
T3 (7.36-32.16 km)	**0.53**	0.46; 0.60	**0.57**	0.50; 0.66	**0.59**	0.52; 0.68
Age			**1.03**	1.02; 1.04	**1.03**	1.02; 1.04
Sex			**2.24**	2.00; 2.52	**2.28**	2.03; 2.56
Education						
≤11 years of education			**0.45**	0.39; 0.53	**0.47**	0.40; 0.55
12-13 years of education			**0.63**	0.56; 0.71	**0.64**	0.57; 0.72
>13 years of education			**-**			
Car ownership			0.90	0.73; 1.12	**0.92**	0.74; 1.14
Energy intake (kcal)			**1.00**	1.00; 1.00	**1.00**	1.00; 1.00

Presence of other food outlets	**1.00**	1.00; 1.00

DASH, Dietary Approaches to Stop Hypertension. Accordance to DASH was defined as a DASH score >28. Coefficients were derived from logistic regression analyses. **Boldface** indicates statistical significance ($p<0.05$). T1 is the tertile with shortest distance to the nearest supermarket (reference group), while T3 is the tertile with the longest distance to the nearest supermarket. Model 1 is an unadjusted model; in model 2 associations are adjusted for individual level covariates (age, sex, car ownership, educational level and energy intake); in model 3 associations are additionally adjusted for exposure to other food outlets within a 1 mile Euclidean buffer of the nearest supermarket

Appendix Figure 1: Visualization of the study area including supermarket locations.

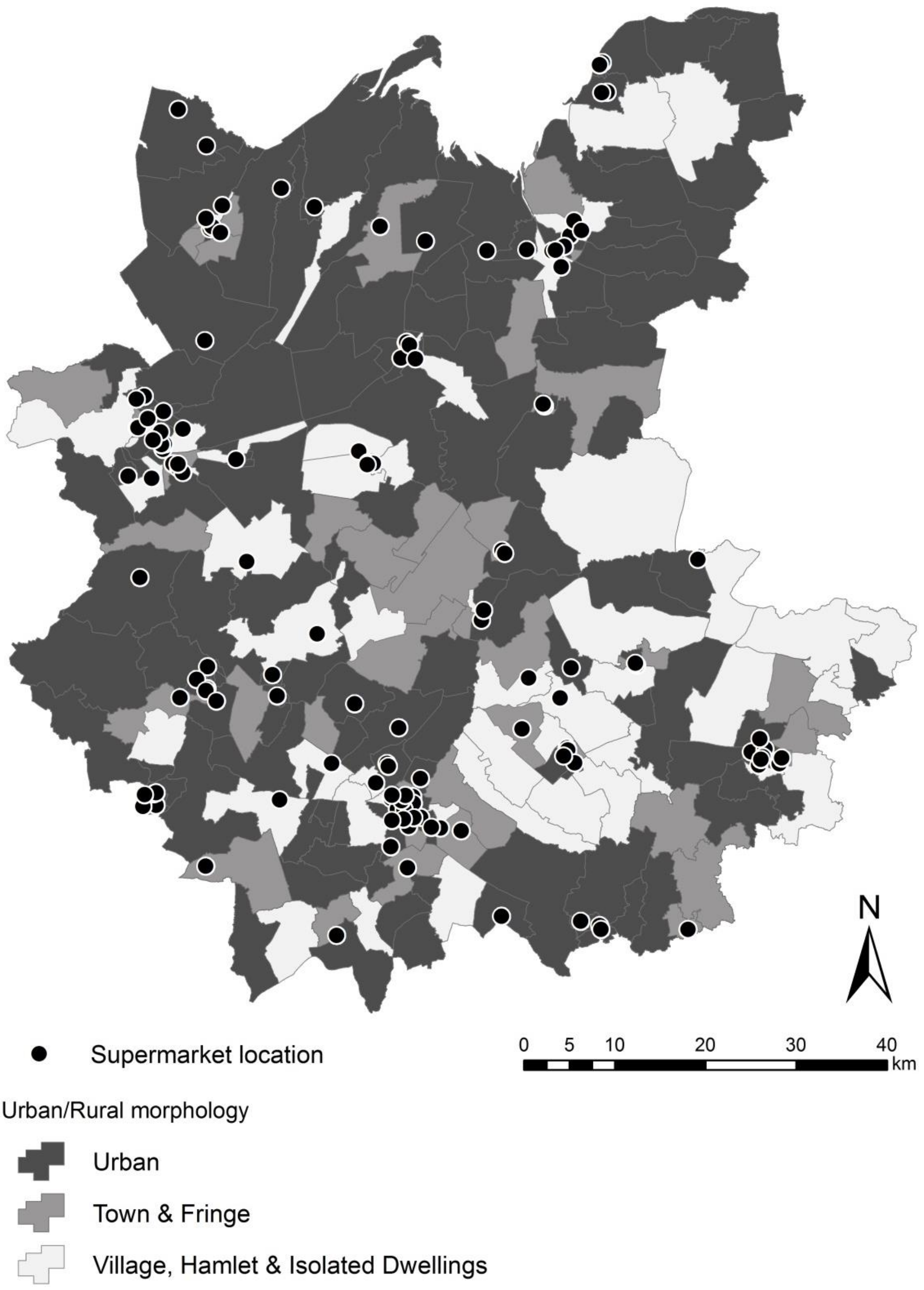

Appendix Figure 2. Visualization of geographic and economic accessibility to supermarkets.

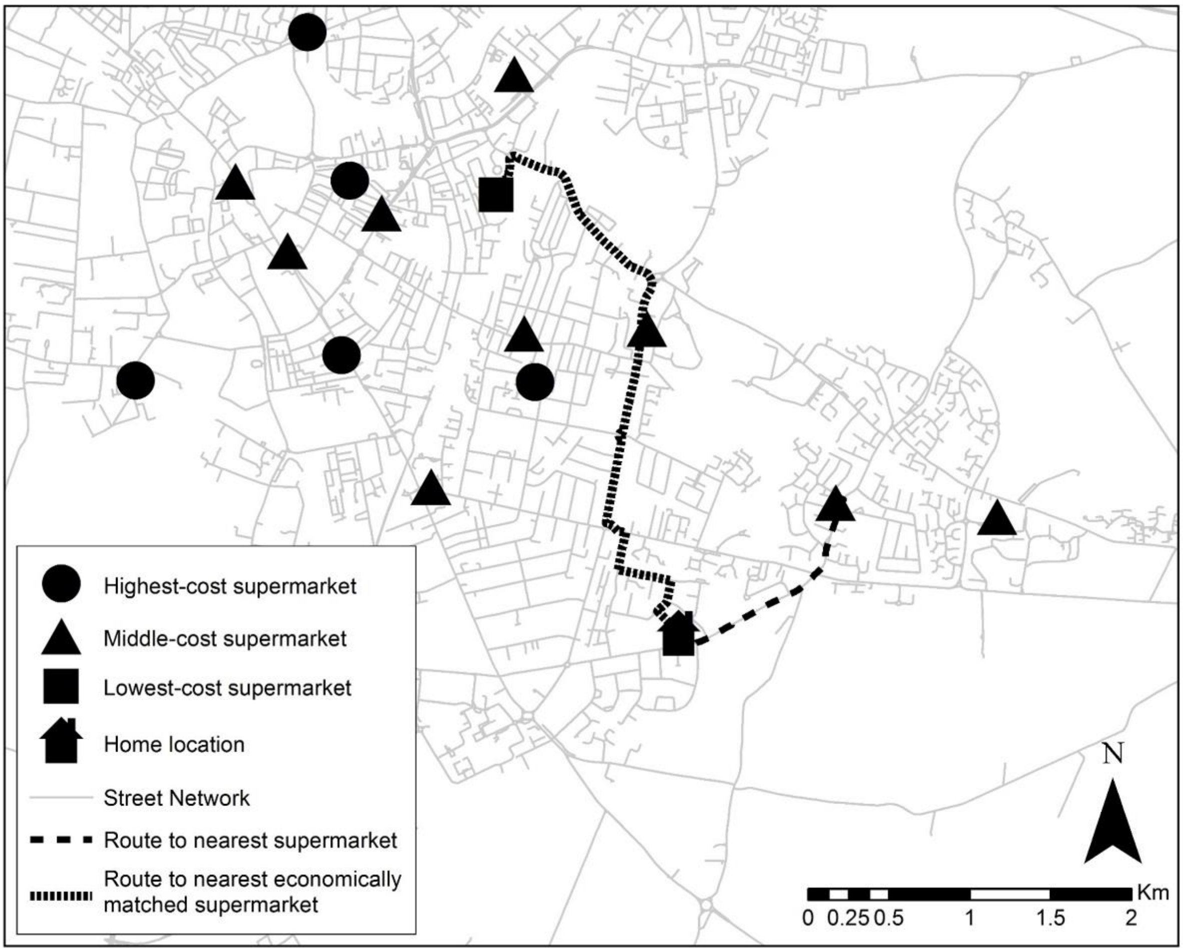

Appendix Figure 3: Participant flow chart for Fenland Study sample restriction, for analyses reported in this study.

Fenland Study Participants

Participants enrolled into study **at time of analysis**: n=11,857

Missing or not geocodeable home address; home address outside study area: n=1656

Provided valid home address: n=10201

Extreme reported values for energy intake based on sex-specific cut-offs: n=273

Provided valid home address and energy intake values: n=9928

Incomplete age, sex, education, dietary quality, smoking status, household size, household income or car ownership data: n=204

Analytic sample: n=9724
